# Vascular Tissue Engineering Using Scaffold-Free Prevascular Endothelial–Fibroblast Constructs

**DOI:** 10.1089/biores.2018.0039

**Published:** 2019-01-08

**Authors:** Sanket Pattanaik, Chase Arbra, Heather Bainbridge, Sarah Grace Dennis, Stephen A. Fann, Michael J. Yost

**Affiliations:** Department of Surgery, Medical University of South Carolina, Charleston, South Carolina.

**Keywords:** angiogenesis, prevascular, scaffold-free tissue engineering, *in vivo* vascularization, endothelial cells, blood vessel, coculture models

## Abstract

Vascularization remains a substantial limitation to the viability of engineered tissue. By comparing *in vivo* vascularization dynamics of a self-assembled prevascular endothelial–fibroblast model to avascular grafts, we explore the vascularization rate limitations in implants at early time intervals, during which tissue hypoxia begins to affect cell viability. Scaffold-free prevascular endothelial–fibroblast constructs (SPECs) may serve as a modular and reshapable vascular bed in replacement tissues. SPECs, fibroblast-only spheroids (FOS), and silicone implants were implanted in 54 Sprague Dawley rats and harvested at 6, 12, and 24 h (*n* = 5 per time point and implant type). We hypothesized that the primary endothelial networks of the SPECs allow earlier anastomosis and increased vessel formation in the interior of the implant compared to FOS and silicone implants within a 24 h window. All constructs were encapsulated by an endothelial lining at 6 h postimplantation and SPEC internal cords inosculated with the host vascular network by this time point. SPECs had a significantly higher microvascular area fraction and branch/junction density of penetrating cords at 6–12 h compared with other constructs. In addition, SPECs demonstrated perivascular cell recruitment, lumen formation, and network remodeling consistent with vessel maturation at 12–24 h; however, these implants were poorly perfused within our observation window, suggesting poor lumen patency. FOS vascular characteristics (microvessel area and penetrating cord density) increased within the 12–24 h period to represent those of the SPEC implants, suggesting a 12 h latency in host response to avascular grafts compared to prevascular grafts. Knowledge of this temporal advantage in *in vitro* prevascular network self-assembly as well as an understanding of the current limitations of SPEC engraftment builds on our theoretical temporal model of tissue graft vascularization and suggests a crucial time window, during which technological improvements and vascular therapy can improve engineered tissue survival.

## Introduction

The field of tissue engineering advances an exciting array of solutions for organ repair and wound healing. However, to realize this potential, several hurdles must be overcome. Vascularization is arguably the most important practical limitation in tissue engineering, imposing both dimensional and time constraints on the technology. For example, tissue-engineered constructs (TECs) designed to replace donor material typically have dimensions exceeding the diffusional limit of nutrients and oxygen, cited in literature as between 100 and 200 μm.^1,2^ This diffusion limit roughly corresponds to the distance between cells in mammalian tissue and the adjacent vascular bed.^3^

To sustain larger TECs, an intact vascular pedicle, consisting of an inlet, outlet, and a perfusable capillary bed, must either preexist or form rapidly. The need for rapid neovascularization or anastomosis of TECs underscores another major knowledge gap in tissue engineering. A systematic approach toward solving this problem should include an appropriate step-by-step timeline of vascularization events for these constructs. This temporal model should illustrate the rate-limiting steps in blood vessel formation that slow vascularization of tissues. Previous literature has tracked vascularization at endpoints past a few days to weeks following implantation.^4–7^ These data are incomplete as they do not evaluate dynamics of vascularization at early time points (hours to a few days) following implantation. Host-derived microvessels can invade an avascular implant as part of the foreign body response, but the process has been reported to take at least a week.^8^ Peri-implant neovascularization in such a scenario typically proceeds shortly after formation of a fibrous capsule.^8^ Unfortunately, ischemic damage to tissues occurs within a time span of hours rather than days,^9^ necessitating a focus on processes impacting vascularization within a 24 h postimplantation window. A systematic temporal model for vascularization of TECs should focus on this time scale, informing us on the degree of vascularization attainable in the hours following implantation. This information will serve as the backbone of future efforts to improve implant vascularization rate.

**FIG. 1.**
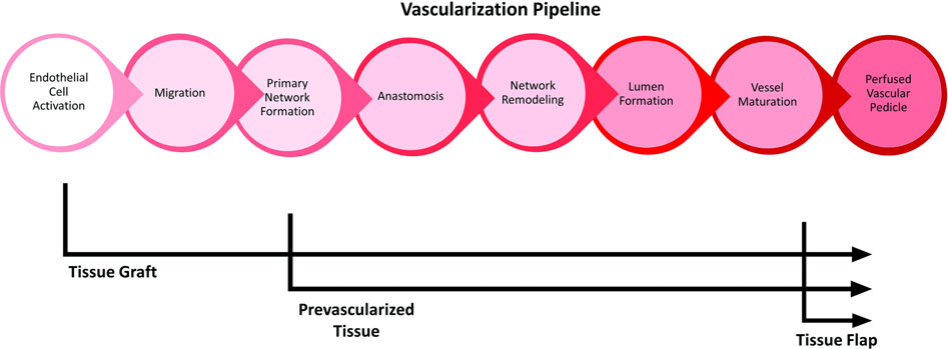
A temporal model of vascularization in TECs. Model accounts for the development time associated with (1) endothelial cell activation, (2) migration of endothelial cells and remodeling of the implant stroma, (3) primitive network formation (4) anastomosis of host/implant endothelial structures, (5) network remodeling (6) lumen formation within the endothelial architecture, and (7) maturation of vessels through recruitment of mural cells. This leads to formation of a blood-perfused vascular pedicle in an implant. Depicted is the expected entry point of different implant types, with preceding development either occurring *in vitro* or supplied by donor. TEC, tissue engineered construct.

Vascularization of an avascular tissue can be modeled by a well-ordered series of events (Fig. 1), starting with endothelial cell activation, network formation, and ending with lumen formation and perfusion of mature vessels.^10^ The kinetics of each phase of the vascularization pipeline can be explored by evaluating TECs that, by design, enter the pipeline at different points. The field of reconstructive surgery provides useful terminology to describe the spectrum of entry points. Restoration of damaged or dysfunctional tissue can either involve transfer of tissue lacking blood flow, termed a graft, or transfer of a tissue sustained by its own blood supply in the form of a vascular pedicle, termed a flap.^11^ Unique to current engineered grafts are the absence of any vascular architecture, forcing them to enter the vascularization pipeline at the earliest time point. The unrealized goal of tissue engineering is the formation of a true tissue flap, where the entire vascularization pipeline can be completed *in vitro*, and perfusion can be established simply through microsurgical anastomosis of mature, perfusable TEC vessels to the host vasculature.

Prevascularization, or *in vitro* assembly of primitive endothelial networks resembling a capillary bed, is one method of attaining a more flap-like TEC design. Laschke and Menger make a useful distinction between angiogenesis and inosculation that explains the utility of prevascularization: angiogenic sprouting, while potentially faster than *de novo* vasculogenesis, is still a slow process, whereas inosculation or merging of microvessels into larger diameter vessels occurs more rapidly.^12^ Specifically, *in vivo* microvessel growth by angiogenic sprouting occurs at a peak rate of 5 μm/h.^12,13^ Spanning an entire implant exceeding dimensions of a few hundred micrometers at this rate is too slow to prevent ischemic damage,^12^ as hypoxia peaks in skeletal muscle at approximately 8 h.^9^ Creating implants *in vitro* with preformed vessels can vastly reduce the distance an angiogenic sprout must travel to bridge the host and implant vascular elements or shortens the entire process to direct inosculation.^14^ In addition, a preformed endothelial network has essentially completed the phases of extracellular matrix (ECM) remodeling and proteolysis characterizing the branching morphogenesis of the vascular network.^15^ Prevascularization approaches have been used in past years by growing an implant, such as artificial skin, around a host arteriovenous fistula that has been extended to an externally located pocket, allowing angiogenic sprouts to permeate the implant.^2^ This surgical approach to forming a vascular pedicle ensures adequate vascularization of an implant, but has a limited practical application because the implant must remain coupled to an immobilized host during its development. More recent methods have involved coculture of human umbilical vein endothelial cells (HUVECs) and mesenchymal stem cells (MSCs) to allow spontaneous formation of primitive vascular network^16,17^; however, translational limitations exist with this technology. The difficulty of regulating differentiation of MSCs as well as their potential tumorigenicity limits the appeal of using these multipotent cells to develop a prevascular stroma.^18,19^ Adipose microvascular cell-derived microvessels appear to be more stable than HUVEC microvessels, suggesting that cell source is another means to improve engraftment potential of endothelial/stromal cell cocultures.^20,21^

We have developed and previously reported on the anastomotic potential of a scaffold-free prevascular implant model that is formed from the coculture of human adipose microvascular endothelial cells (HAMECs) and normal human dermal fibroblasts (NHDFs).^5^ We use primary human adipose-derived endothelial cells to ensure clinical translatability, where an autologously derived population of cells can be reimplanted in a patient with minimal morbidity due to immune compatibility complications. The adult fibroblasts create the extracellular-rich stroma necessary to support a vascular bed and provide additional proangiogenic stimuli.^22^ Mature endothelial cells, while capable of forming spontaneous capillary-like tubes *in vitro*, appear to require consistent input of proangiogenic environmental signaling. Fibroblasts, through constitutive expression of vascular endothelial factor (VEGF), basic fibroblast growth factor, and angiopoitin-1 (Ang-1), address this basic need.^22^ The presence of fibroblasts corresponds to an increased microvessel density within an implant and stabilization of vessels by signaling endothelial cell to express smooth muscle actin.^16^ Other ECM proteins deposited by the fibroblasts such as laminin, collagen type I, and collagen type IV are needed for vessel maturation.^10^ We have previously reported that a specific 1:4 ratio of human microvascular endothelial cells and fibroblasts maximizes the density of endothelial cords when allowed to self-assemble in a scaffold-free nonadherent environment. Increasing the density of fibroblasts resulted in endothelial clusters without cords, and increasing density of endothelial cells resulted in structures lacking avascular stromal areas consistent with a vascular bed.^23^ Within 3 days of *in vitro* culture in an agarose mold, the coculture generated an ECM containing laminin, type I collagen, and fibronectin. The interplay between ECM components, such as laminins and fibrillar collagen, and cell surface integrins plays a key role in vascular lumen formation.^24^ Czajka et al. noted the presence of laminin throughout the ECM rather than constrained to the basement membrane, as would be expected in mature blood vessels. This suggests a more embryonic, primitive vascular network.^23^ Implanted constructs show early signs of anastomosis in a rat hind limb muscle. Within 3 days, there was evidence of red blood cell perfusion in the implants with vascular structures that persisted out to 2 weeks.^5^

The SPECs retain a set of properties that could have inherent therapeutic value when incorporated into replacement tissue technologies. SPEC spheroids can readily fuse to form larger constructs in unconstrained nonadherent conditions and can reshape their cytoskeletal structures to assume patterns dictated by confinements such as an agarose mold.^25^ This scalable and shapeable nature, coupled with the primary endothelial cord networks of the SPECs, makes them ideal analogs to a vascular stroma or artificial vascular bed that is inherent to the function of most tissues. The SPECs can readily incorporate renal segments^26^ and pancreatic islets,^27^ paving the way for rapidly vascularizable artificial renal grafts and bioartifical pancreas. To make these technologies a reality, however, we must first identify processes that speed or hamper anastomosis and perfusion of implanted grafts.

In this study, we contrast the vascularization behavior of an avascular graft-like fibroblast spheroid and vascularization of our more flap-like prevascular implant model, offering two different points of entry into our proposed temporal model of vascularization. While previous examples of prevascular engineering demonstrated successful anastomosis in a time span of weeks, few studies have taken a systematic stepwise approach to temporally assess vascular development beginning at a few hours following implantation of engineered tissue. In this study, we examine early steps in vascular development following implantation of our novel scaffold-free, prevascular endothelial–fibroblast tissue-engineered constructs (SPECs). The SPEC model permits examination of the transition from primitive network to a complex ordered anastomosed network. Specifically, we hypothesize that the existing self-assembled primitive network of the SPECs allow earlier host-implant anastomosis and increased presence of lumen-containing vessels in the interior of the implants by 24 h compared to avascular grafts such as fibroblasts spheroids.

## Materials and Methods

### Cell culture

HAMEC (ScienceCell, Carlsbad, CA, 7200) were cultured in endothelial growth medium-2 (EGM2) (Lonza, Allendale, NJ; CC-3156 & CC-4176). Normal human dermal fibroblasts-Adult (NHDF-Ad) (Lonza; CC-2511) were cultured in fibroblast growth media-2 (FGM2) (Lonza; CC-3131 & CC-4126). Cells were collected between passages 7–10 for use in experiments and implant development.

### *In vitro* implant development

The scaffold-free prevascular endothelial fibroblast constructs (SPECs) were modifications of a protocol established by Czajka and Drake.^23^ Rod-shaped troughs of 0.9 cm by 0.1 by 0.5 cm depth were constructed in 2% UltraPure™ Agarose (Invitrogen, Carlsbad, CA, 16500-100) and high density 4:1 mixtures of 720,000 NHDF-Ad cell, and 180,000 HAMECs were pipetted into the troughs. Cells were then cultured in a 2:1 mixture of FGM2 and EGM2 for 3 days. Implants were either collected in Dulbecco's phosphate-buffered saline for use in surgical implantation or collected and fixed in 4% paraformaldehyde for histology. Fibroblast-only spheroids (FOS) were constructed similarly with 900,000 NHDF-Ad and no HAMECs. Silicone fragments of rectangular box dimensions of 0.6 cm by 0.1 cm by 0.1 cm (to match the eventual dimensions of the cell-based rods) were autoclaved and stored in DPBS in preparation for implantation.

### Surgery

Animal procedures were conducted following approval by the Institutional Care and Animal Use Committee (IACUC) of the Medical University of South Carolina. Fifty-four Sprague Dawley rats (Charles River Labs, Wilmington, MA) were divided into three groups: SPECs (*n* = 15), FOS (*n* = 15 rats), and silicone implants (*n* = 15 rats). Nine rats were set aside for sham surgeries. Surgeries were performed as described by Calder and colleagues.^5^ Implants were placed in submuscular pocket, with the long axis oriented parallel to the hind limb running proximal to distal. Five rats within each implant group were sacrificed at 6, 12, and 24 h, with muscle excised from the left hind limb *en bloc* with implant or sham surgery, placed in O.C.T. compound (Tissue-Tek 4853, Torrance, CA), and frozen at −70°C for cryosectioning. Muscle from the right limb was harvested for comparison.

### Histology

Tissue sections were fixed in 4% paraformaldehyde solution for 30 min and subjected to hematoxylin and eosin staining, direct or indirect immunofluorescence labeling. Tissue sections were directly labeled with Hoechst 33342 nuclear stain (Molecular Probes, 1:10,000), and Alexa Fluor™ phalloidin 488 (ThermoFisher Scientific A12379, 1:500) for f-actin. Selected sections were stained with primary antibodies to von Willebrand Factor (Abcam; Catalog# ab6994, 1:1000), CD31 (Abcam ab28364, 1:50), human CD31 (monoclonal antibody) (R&D Systems BBA7; 1:25), and smooth muscle actin (ThermoFisher Scientific PA5-19465; 1:1000). Primary antibodies were fluorescently tagged with the secondary antibodies Alexa Fluor goat anti-mouse 488, goat anti-rabbit 546, goat anti-mouse 546, and goat anti-rabbit 633 (ThermoFisher Scientific; A-11001,11035,11030,21070, 1:500). Sections were mounted on Colormark Plus microscope slides in Prolong Gold antifade reagent (Molecular probes P36934).

### Western blot

SPECs and FOS were collected after 1, 2, and 3 days of culture in 2% linear agarose molds as previously described. Samples were snap frozen and mechanically homogenized in RIPA lysis buffer with protease inhibitor cocktail. Samples were maintained in constant agitation for 2 h at 4°C and centrifugated for 20 min at 16,000 *g* at 4°C. Supernatant was stored in fresh tube at −20°C. Pierce™ BCA Protein Assay Kit (ThermoFisher Scientific 23227) was used to estimate protein concentration for samples as per manufacturer's instructions. Before gel electrophoresis, samples were diluted in RIPA buffer to attain 20 μg of protein in 20 μL solution, and further diluted 1:1 in 2 × Laemmli Sample buffer to attain 40 μL loading volumes. Samples were loaded onto Any kD™ Mini-PROTEAN^®^ TGX™ Precast Protein Gels. Following protein separation and overnight transfer onto PVDF membranes, western blots were performed using antibodies toward GAPDH (loading control) (Calbiochem CB1001; 1:1000), VEGFR2 (Abcam; ab39256, 1:900), VE-Cadherin (ThermoFisher Scientific 36-1900; 1:250), vWF (Abcam; ab6994, 1:500), and DLL4 (Abcam; ab7280, 1:1000).

### Microscopy and quantitative analysis of endothelial cord organization

Confocal images were acquired using Leica TCS SP5 AOBS Confocal Microscope system (Leica Microsystems, Inc., Buffalo Grove, IL) and collected as average projected z-stacks at 20 × and 40 × magnifications. Stitching was performed using LAS AF v2.6.3 Build 8173 and encompassed the entire visible cross section of each implant. Images were auto-enhanced and analyzed using ImageJ (NIH, Bethesda, MD). To analyze endothelial cord organization, stitched 40 × (512 × 512) confocal images of sections stained with antibodies for vWF and CD31 were segmented as follows. Average projected images (10 μm depth) were autothresholded in ImageJ (Otsu auto threshold^28^) and binarized. Perimeter of the implants and endothelial capsule around implants were drawn freehand. Binary images were used to calculate microvessel area fractions of the implant. Binary images were skeletonized using an ImageJ plugin, provided by Arganda-Carreras et al.,^29^ with branches pruned by lowest intensity voxel. Resulting images were used to calculate number of junctions and vessel branch lengths.

### Statistical analysis

Statistical Analysis was performed using IBM SPSS for Windows Version 24.0 (Released 2017, Chicago, SPSS, Inc.). Levene's test for homogeneity of variance was used to determine equality of variances (α = 0.05). For data with equal variance between groups, one-way ANOVA was performed with *post hoc* application of Bonferroni's *t* test used to compare microvessel density, degree of penetrance of vessels, and branching density among the three implant types at each time point. For data with unequal variances, Welch's one-way ANOVA was performed with Dunnett T3 *post hoc* corrections.

## Results

**FIG. 2.**
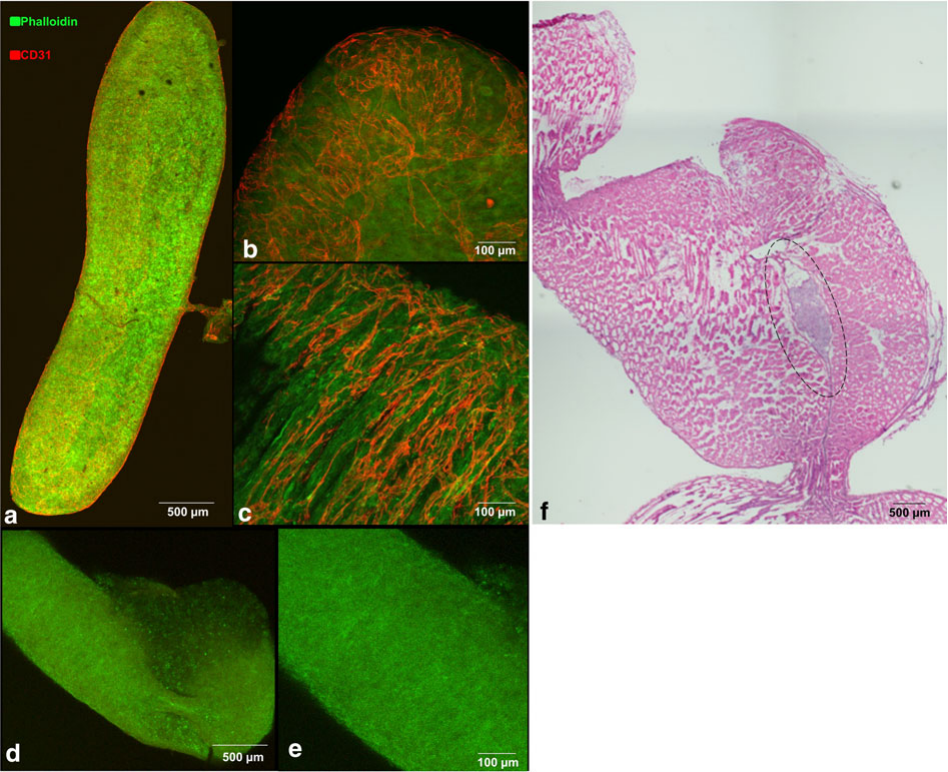
**(a–c)** Whole-mount immunofluorescence image of rod-shaped SPEC liberated from 2% agarose mold. F-actin fibers present in fibroblasts labeled with Phalloidin 488 (green) represent the stromal components of the implant, and CD31-labeled structures (red) represent capillary-like cords of endothelial cells. **(d, e)** Whole-mount immunofluorescence images of rod-shaped FOS similarly presented with F-actin fibers in fibroblasts labeled with Phalloidin 488 (green). However, these constructs lack presence of CD31-labeled structures (red) **(f)** Light microscopy images of hematoxylin and eosin-stained rat hind limb tissue sections (10 μm) containing cross section of implanted SPEC (encircled) between vastus lateralis and biceps femoris muscles. FOS, fibroblast-only spheroids; SPEC, scaffold-free prevascular endothelial–fibroblast construct.

The linear rod-shaped SPEC implant resulted in an avascular space composed of f-actin presenting fibroblasts and CD31^+^ endothelial cells (Fig. 2). Endothelial cells resembled loose, filamentous cords (∼2–3 μm thick) without an obvious hierarchy of larger and smaller vessels. The microvessel area fraction of the implants, or percentage of implant occupied by endothelial structures before implantation, was approximately 28% ± 13% (Fig. 2), ranging between a third and a fourth of the total implant volume. The implanted constructs were localizable within the rat submuscular pockets by the cell tracker in immunofluorescence images and seen as basophilic regions within the eosinophilic musculature in hematoxylin and eosin images. Pinch-marks made by forceps were used as fiducial points to localize implant during sectioning (Fig. 2).

**FIG. 3.**
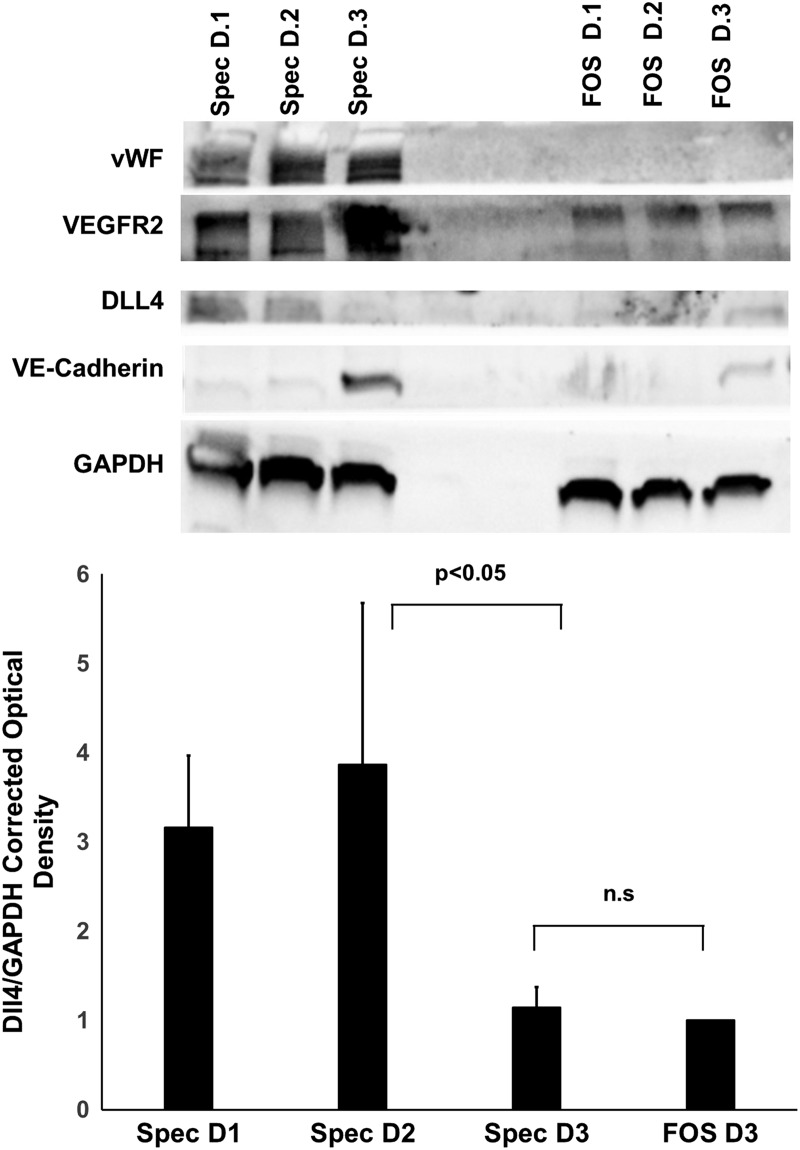
Western blot of vascular markers VEGFR2, VE-cadherin, vWF, and DLL4 of SPEC and FOS during *in vitro* assembly. GAPDH house-keeping protein functioned as the loading control. Anti-Dll4 blotting corresponding to endothelial tip cell phenotype was tracked across day 1, 2, and 3 of SPEC and FOS growth. Relative optical density was calculated by normalizing to FOS day 3 controls. Ratio of relative optical density for DLL4 to the relative optical density of GAPDH is displayed (*n* = 3). Dll4 peaks at D2 and decreases at D3 are similarly reflected by VE-cadherin western blot. This suggests a period of quiescence after 2 days incubation of the SPECs. VEGFR2 and vWF, markers of endothelial cells, remain stable after reaching a peak at D2, suggesting limited endothelial cell proliferation at D3. ns, no statistical significance.

Before implantation, the SPECs presented with vWF, VEGFR2, and VE-cadherin as indicated by (Fig. 3). Control FOS implants displayed negligible levels of these vascular markers. Western blotting for DLL4, a marker of endothelial tip cell phenotype, showed increased levels at days 1 and 2 of SPEC incubation, but decrease by day 3 compared to FOS controls. A time-series image of GFP-tagged HAMECs within the SPECs placed on a tissue culture plate demonstrated a high degree of motility of the endothelial cells following 3 days of culture, with some cells tracked as traveling over 400 μm in distance within a 24 h time series (Supplementary Fig. S1).

**FIG. 4.**
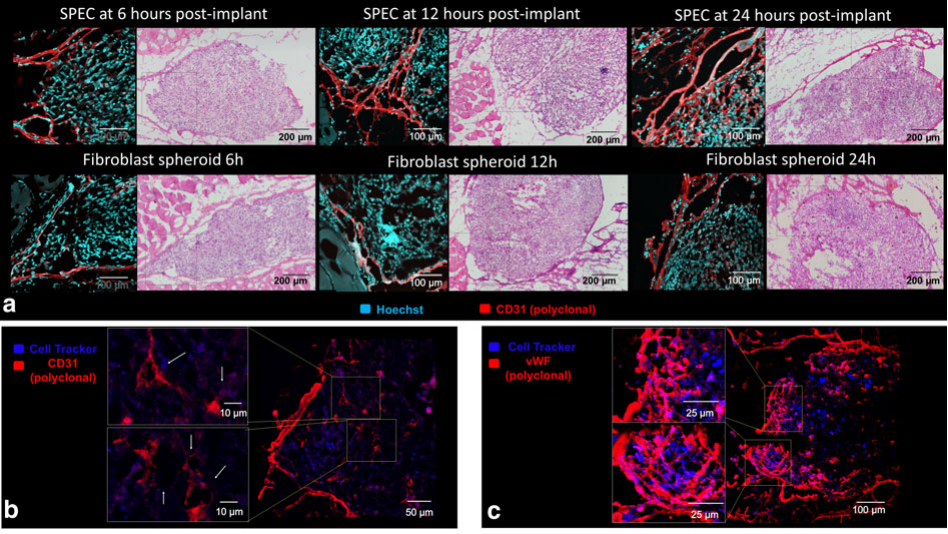
**(a)** Endothelial reorganization within TECs was imaged through immunofluorescence imaging with Hoechst nuclear stain (Cyan) and anti-CD31 antibody stain for endothelial cells (Red). A capsule-like layer of endothelial cells surrounds both construct types at 6 h. The internal endothelial structures in the SPEC interdigitate with this capsule, resulting in a lacy layer of cords. By 12 h, coalesced bands of endothelial cords penetrate SPEC interior, while fibroblast spheroids still lack internal endothelial structures. At 24 h, both fibroblast spheroids and SPECs are invaded by endothelial cords, with SPECs containing a more complex branching structure at the implant/muscle interface. **(b)** CD31^+^ structures (red) with apparent lumens are visible within implanted SPEC cells labeled with cell tracker (blue) at 12 h postimplantation. **(c)** Invading von Willebrand factor+ endothelial branches from the endothelial capsule inosculate with cell tracker positive endothelial cords (magenta) indicating both host and implant contribution to SPEC vascular network.

**FIG. 5.**
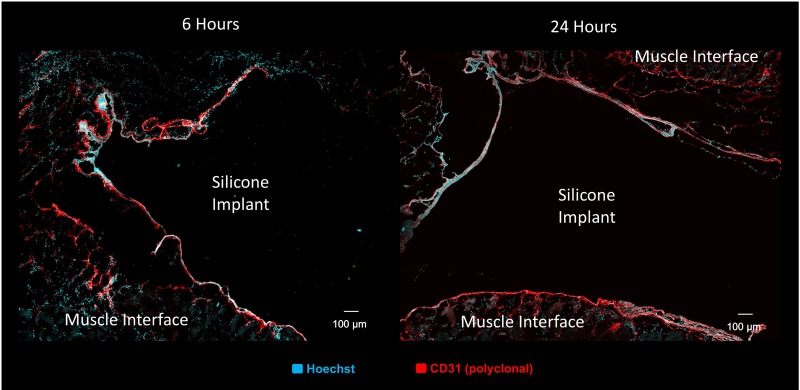
Immunofluorescent images of tissue sections containing silicone implant cross sections (10 μm) were labeled with Hoechst nuclear stain (cyan) and CD31 antibody stain for endothelial cells (red). Sections indicate presence of endothelial-rich capsule (red) surrounding implants but lack of any structures or cells permeating the implants themselves at 6 and 24 h postimplantation.

All three implant types developed CD31- and vWF-positive capsules as early as 6 h postimplantation, indicating early endothelialization of the host-implant interface (Figs. 4 and 5). The SPECs showed this capsule interdigitating with internal vascular elements at 6 h; however, neither the FOS nor the silicone showed endothelial cords within the implant interior at the 6 h time point. By 12 h, the SPECs displayed larger vessel-like bands composed of smaller cords penetrating through the implants, some of which bisected the implants (Fig. 4a). By 24 h, many small lumen-like structures were present at the periphery of the implant connecting with the thicker endothelial capsules (Fig. 4a). Penetrating cords within the SPECs colocalized with the cell tracker (Fig. 4c) indicate implant contribution to these endothelial structures.

**FIG. 6.**
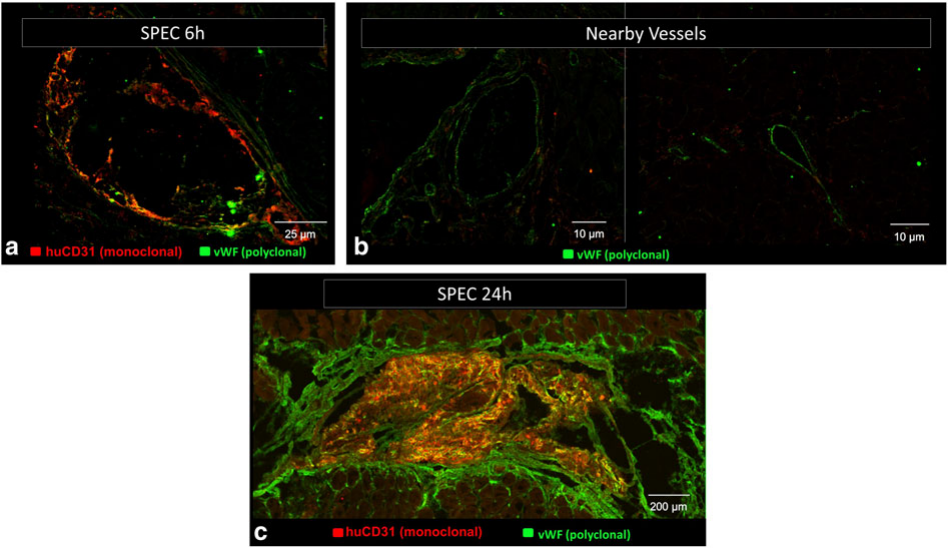
**(a–c)** Monoclonal anti-human CD31 antibody stain (red) and polyclonal anti-vWF antibody stain (green) colocalize in implant-derived human endothelial structures (yellow). These structures are limited to the interior component of the implanted SPECs at both 6 and 24 h. Host-derived vascular networks only stain for anti-vWF stain and are visible at the external capsule surrounding the SPECs and in vessels distal to the implant site in the opposite host hind limb muscle.

**FIG. 7.**
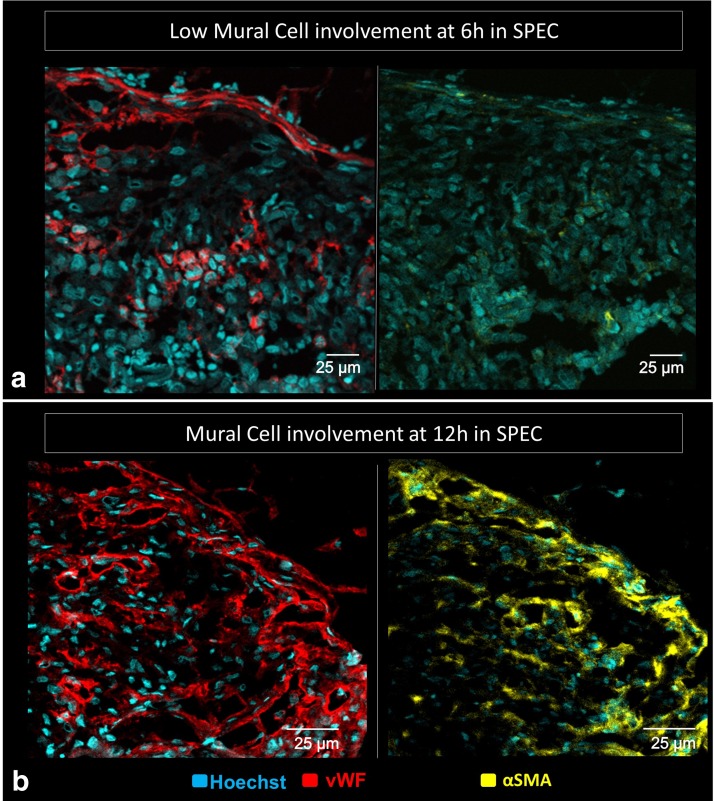
Immunofluorescent images of implanted constructs compares vWF^+^ vessels (right; red) and αSMA^+^ cells (left; yellow) on sister sections (10 μm apart). **(a)** SMA^+^ expression at 6 h postimplantation is not consistently associated with vascular development with few tubule-associated SMA^+^ cells. **(b)** At 12 h postimplantation, sister sections demonstrate presence of αSMA^+^ cells in regions presenting with vWF^+^ vessels with apparent lumens. This is consistent with recruitment of αSMA^+^ pericytes or vascular mural cells expected during vessel maturation.

The internal vasculature of the SPECs was largely implant derived, as indicated by the monoclonal human CD31 stain (Fig. 6). By contrast, the endothelial capsule components in the SPEC and FOS only express polyclonal vWF, which labels both rat and human endothelial cells, indicating derivation from the rat hosts. Vessels in control sections of muscle without a surgical pocket similarly solely express vWF in their lumens. The SPECs displayed SMA^+^ puncta indicative of pericyte involvement^30^ as early as 6 h, with vessel-like patterns matching vWF expression on sister sections by 12 h (Fig. 7).

Cells within both FOS and SPEC continued to die between 6 and 24 h as evidenced by increased TdT dUTP Nick-End Labeling (TUNEL^+^) staining for apoptotic and necrotic cells (Supplementary Fig. S2). There was no significant difference between FOS and SPEC survival at 6–24 h. This corresponds to a lack of evident injected dye perfusion of vessel lumens within the 24 h window despite presence of red blood cells in SPEC implants from tissue harvested at 24 h (Supplementary Fig. S3).

## Discussion

The timeline of vascular events (Fig. 1) immediately following implantation of prevascular tissue is crucial to evaluating the technology. The observation windows selected in this study were designed to dissect the components of endothelial organization, cord sprouting, anastomosis, network remodeling, lumen formation, and, ultimately, vessel maturation that occurred early in the *in vitro* development of our implant and shortly following implantation. The 6–12 h postimplantation window is particularly important as it contains the time points associated with a peak in markers of hypoxic stress found within autologous full-thickness muscle flap transplants in earlier studies by our laboratory.^9^

### *In vitro* SPEC development was consistent with nonrandom organization of a proangiogenic vascular network

The endothelial cord formation within the SPECs is a nonrandom process, with the initial dispersed endothelial cells coalescing into cords throughout the 3-day incubation period. This migration stands contrary to the popular theory of cellular behavior termed the differential adhesion hypothesis.^31^ If the rearrangement was entirely driven by passive cell adhesion behavior rather than active vascular development processes, a single interface between endothelial cells within the core and fibroblasts on the periphery would be observed. This behavior would optimize interfacial energy based on cell type-specific expression of adhesion molecules such as cadherins.^32^ Further evidence of active vessel formation within the implant is provided by western blot data, through expression of vascular markers such as VEGFR2, VE-cadherin, and vWF. Dll4 expression in implants is consistent with angiogenic and anastomotic potential of endothelial cells as reported in literature.^33,34^ The upregulation of DLL4 expression in SPECS at D2 is consistent with increased vascularization of the implant; in contrast, the comparative downregulation at D3 of incubation, coinciding with when the implant finishes resolving into a solid structure, is consistent with quiescence of the prevascular networks. This period of quiescence may contribute to the latency between anastomosis and *in vivo* tubulogenesis. While ideally anastomosis of prevascular implants should only involve inosculation of externally located cords to the nearby host vasculature, the need to ramp up the angiogenic machinery of the construct cells might delay further morphogenesis of these tubes and delay perfusion through the resulting networks.

### Early 6 h postimplantation period demonstrates rapidity of endothelial capsule formation around implants, and rapid inosculation of scaffold-free prevascular constructs to host

One of the major goals of vascular tissue engineering is near instantaneous perfusion of well-organized cords either by spontaneous *in vivo* inosculation or surgical anastomosis.^1^ While these constructs were not well perfused during the 24 h observation time (Supplementary Fig. S3), endothelial structures extended continuously from the host to the interior of the SPEC implants within 6 h, indicating rapid mobilization of endothelial cells to and from the implant. Notably, the SPEC internal structures are derived from human endothelial cells (Fig. 6), suggesting that this anastomotic network contains, at least, in part, the preformed primitive network that was developed *in vitro*.

**FIG. 8.**
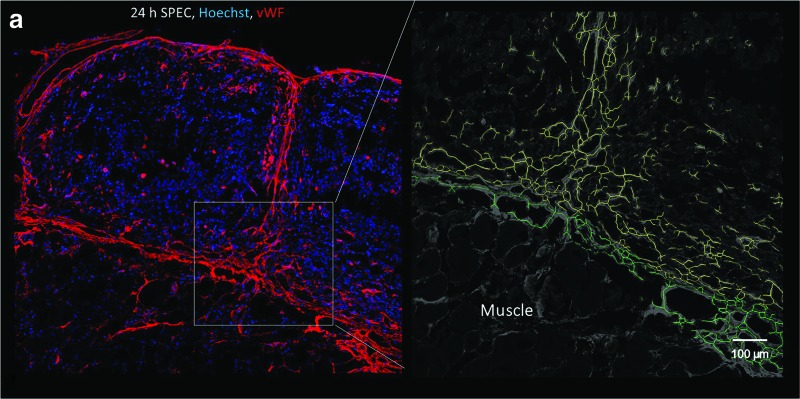

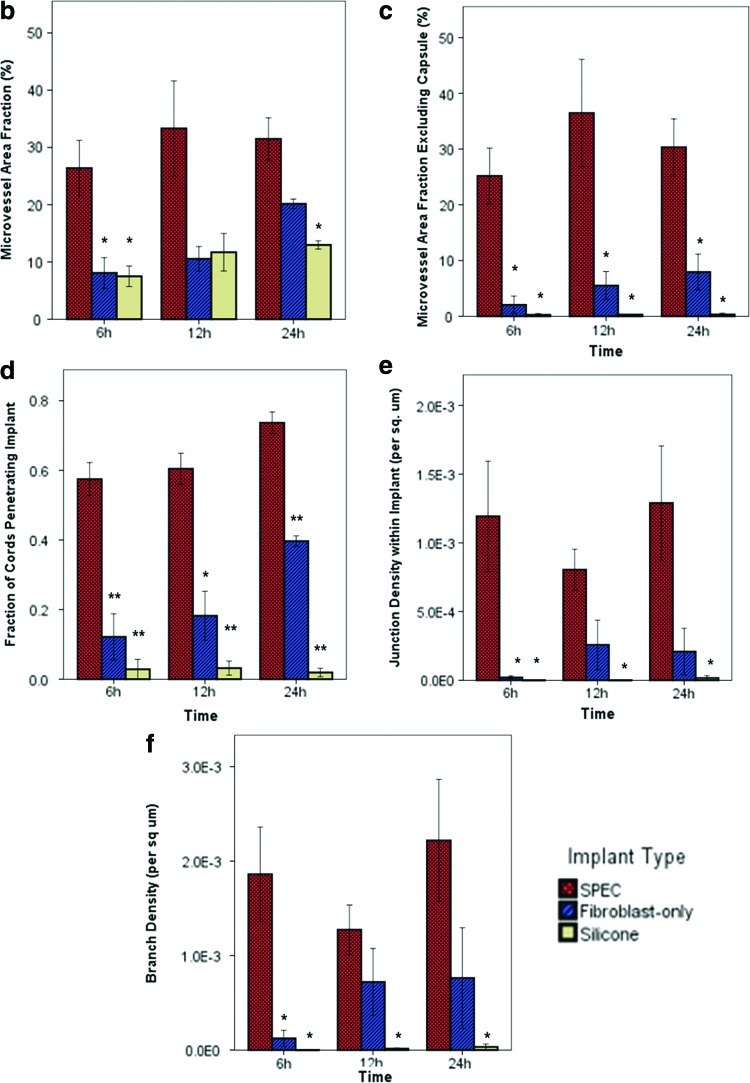
**(a)** Implant vascular structures (CD31 or vWF^+^) were segmented based on immunofluorescent images of tissue cross sections (10 µm depth) containing the entire implant cross section as well as an intact muscle/implant interface. Endothelial structures in direct connection with this interface and the endothelial capsule surrounding the implant were segmented separately (green) from the vascular structures found within the interior of the implants (yellow). **(b)** Total microvessel area fraction, or the percentage of the implant cross sectional area containing vascular elements, was calculated for each implant, with comparisons made between implant types at the 6, 12, and 24 h time points. **(c)** Microvessel area fraction excluding the endothelial capsule at the muscle/implant interface was calculated for each implant. **(d)** The fraction of endothelial cords that penetrate the implant interior was calculated by dividing the length of the cords found excluding the capsule vessels by the total length of the vascular network. This fraction is a surrogate marker of the invasiveness of the vessels within and surrounding each implant within the host. **(e, f)** Junction density was calculated as the number of vessel branch points found per µm^2^ of the implant cross sectional area. Similarly branching density was calculated as the number of branches per µm^2^. These two metrics assess the branching complexity of the developing vascular networks in each implant type across time. Microvessel area, junction density, and branch density of SPECs remain significantly elevated compared to other implants at all time points. Fibroblast spheroids demonstrated the most growth in terms of microvessel area and penetrating tubule fraction between 12 and 24 h with branch density resembling the SPECs at 24 h. *Statistically significant difference (*p* < 0.05) between implant type and SPEC. **Statistically significant difference (*p* < 0.001) between implant type and SPEC.

In addition, the microvessel vascular area of the SPECs 6 h postimplantation is 26% ± 5%, which is comparable to the vascular density of the implant before implantation and only approximately 1.2-fold lower than the average microvessel vascular area fraction throughout the 24 h time point (Fig. 8). The filamentous net-like primordial form of the network before implantation is preserved at 6 h postimplantation, with a high branch point density of approximately 1.2 × 10^5^ per mm^2^ implant tissue, resembling the preimplantation average branching density of 1.12 × 10^5^ per mm^2^. The FOS, on the contrary, show a significantly lower presence of branching endothelial structures within the implant stroma, with most of the 8% ± 3% microvessel area confined to the external capsule (Fig. 8). As such, the fraction of the vascular area that includes internally penetrating tubules in the SPEC is 4.7-fold higher than the fraction within FOS. Surprisingly, endothelial capsule formation occurs around our silicone-based implant, suggesting that the mobilization and reorganization of endothelial structures might be driven by recognition of a foreign body interface more so than communication with the living cells of the implant. The silicone implant displayed essentially no visible microvessels within its interior during the 24 h observation window (Fig. 5).

### The 6–12-h window shows increase in microvessel area in FOS and silicone implants, but not in SPECs; SPECs show remodeling and fusion of existing branches

The 6–12-h window is a period of remodeling in both the SPEC and FOS implant models. Notably, the band of host-derived endothelial structures around the implant appears to thicken with a small but significant increase of internally penetrating branches within some of the FOS. However, the mean microvessel area of the fibroblast spheroids, including the capsular components, remains 3.2-fold lower than the SPEC implants and comparable to that of the silicone implants. In other words, the lack of an existing internal endothelial network in the FOS results in a 12 h latency in vascular development of these implants compared to SPECs. The SPECs, on the contrary, maintain a nearly constant mean vascular area; however, there is a 1.5-fold decrease in junctions within the implant and a two-fold decrease in junctions within the endothelial capsule. This may be attributed to increased condensation of endothelial branches to larger structures, an example of which is seen in Fig. 4, where a denser band of endothelial structures appears to pass through the center of the implant and lumen-like structures begin to appear within the SPEC implant cross sections. This cohesion of existing endothelial cords to form larger multicellular structures is most consistent with formation of the early vascular tree during embryological vasculogenesis.^35^

### Parity between SPEC and FOS angiogenic development by the 24-h time point

By 24 h, SPECs and FOS begin to resemble each other in terms of endothelial organization and mean vascular area, with a greater preponderance of penetrating endothelial cords in the FOS than at previous time points. The advantage in anastomosis provided by the SPECs, thus, seems to lessen at the 24 h time point, as cords from the peripheral endothelial capsule appear to reach the center of the FOS. This rapid invasion of vessels in a previously avascular space is itself a surprising finding. Vascular network can invade on its own as a part of foreign body response, but the process has been cited to take a few days to a week.^1^ The presence of a branching vascular architecture, however, still seems largely limited to the SPECs.

### Evidence of maturation of SPECs at 12–24 h without perfusion

In angiogenesis, maturation of vessels follows anastomosis and usually occurs concurrent with perfusion of vascular networks.^36^ However, in the absence of consistent perfusion, the SPECs show some indication of vessel maturation. Smooth-muscle actin presenting cells, representing mural, stabilizing cells such as pericytes around capillaries, or smooth muscle cells around larger arterioles and arteries, are recruited in the latter stages of angiogenesis, involving a careful interplay between basolateral elements of endothelial cells such as Tie-2, macrophages, and pericytes.^37,38^ The SPECs, which present with an apparently disorganized SMA^+^ cells at the early 6 h time points, show SMA^+^ cells more fully organized around lumen-like structures in the SPEC at the 12 and 24 h time points (Fig. 7).

Perivascular organization alone, unfortunately, does not translate directly to improved vessel patency, as evidenced by poor dye perfusion and leaky vasculature within the implant at 24 h (Supplementary Fig. S3). In addition, both the SPECs and fibroblasts lose approximately 13–14% of their inner cell mass (<4% dead cells at 6 h to 17–18% at 24 h postimplantation), without a statistically significant improvement of cell survival in the SPECs at this time point. Improved survival and functional recovery of implanted tissue will depend on specifically addressing rate limiting steps, both spatially and temporally, at the host-implant interface.

## Concluding Remarks

As past studies have reported inosculation of prevascular implants at 2–5 days,^39^ vascularization dynamics in literature have not focused on early time points preceding a few days following implantation. Our study reveals that an earlier observation window informs us on the relative rapidity of endothelialization around cellular constructs and reveals that the crucial advantages to a prevascular network might be best seen within 6–12 h of implantation. By this period, the groundwork for a vascular pedicle feeding the implants has already been laid, with evidence of reorganization toward a more mature host-implant vascular network. Vascular tissue engineering strategies that proceed from this point should promote lumen formation and patency of the existing vascular architecture.

## Supplementary Material

Supplemental data

Supplemental data

Supplemental data

## Abbreviations Used

Ang-1: angiopoitin-1
ECM: extracellular matrix
EGM2: endothelial growth medium-2
FGM2: fibroblast growth media-2
FOS: fibroblast-only spheroids
HAMEC: human adipose microvascular endothelial cell
HUVEC: human umbilical vein endothelial cells
MSC: mesenchymal stem cells
NHDF: normal human dermal fibroblast
NHDF-Ad: normal human dermal fibroblasts-Adult
SPEC: scaffold-free prevascular endothelial–fibroblast construct
TEC: tissue engineered construct
VEGF: vascular endothelial factor
