# Supplementary material for: Vascular Tissue Engineering Using Scaffold-Free Prevascular Endothelial–Fibroblast Constructs
Source: Biores Open Access. 2019 Jan 8;8(1):1–15. doi: 10.1089/biores.2018.0039 (PMC6327854; doi:10.1089/biores.2018.0039)
Supplement: Supplemental data [file Supp_Fig1.pdf]

Supplementary Data

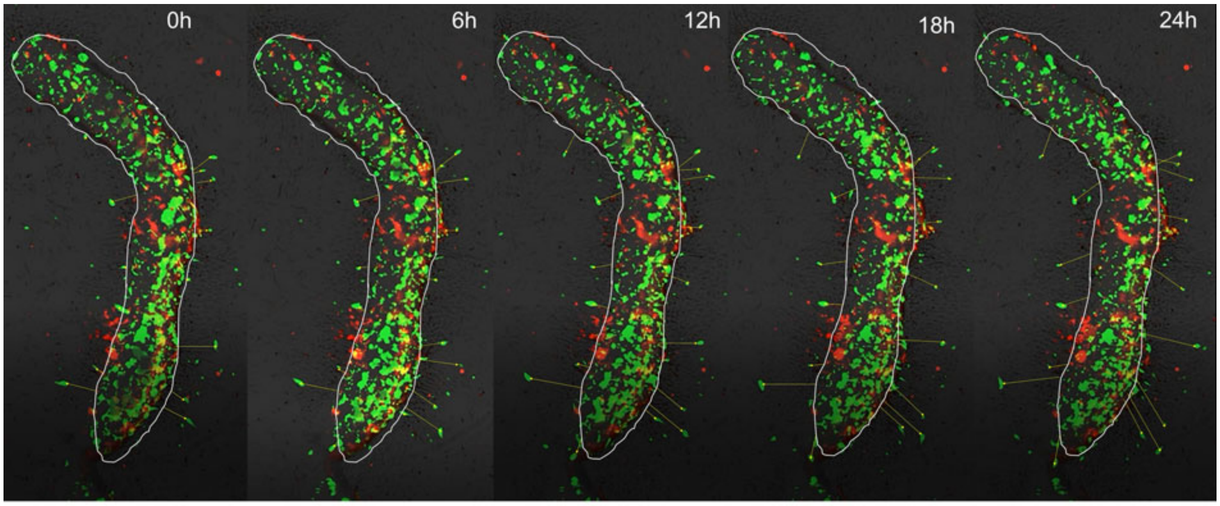

**Average 24 hr Displacement of HAMECs**	247 μm
**Range**	62-494 μm

SUPPLEMENTARY FIG. S1. SPECs with GFP-tagged HAMEC endothelial cells were placed within on a 35-mm dish. Plates contain a monolayer of fibroblasts with an exclusion region created by placement of silicone disc. SPEC were placed within this exclusion zone and imaged using Lionheart Fx live cell widefield microscope. Endothelial movement to and from implant exterior was tracked for three days. The time series depicted includes Day 2 0 h to Day 3 0 h. Magnitude of net displacement vectors were calculated, with an average displacement of 247 μm within a range of 62–494 μm. HAMEC, human adipose microvascular endothelial cell; SPEC, scaffold-free prevascular endothelial-fibroblast construct.
