# Supplementary material for: Vascular Tissue Engineering Using Scaffold-Free Prevascular Endothelial–Fibroblast Constructs
Source: Biores Open Access. 2019 Jan 8;8(1):1–15. doi: 10.1089/biores.2018.0039 (PMC6327854; doi:10.1089/biores.2018.0039)
Supplement: Supplemental data [file Supp_Fig2.pdf]

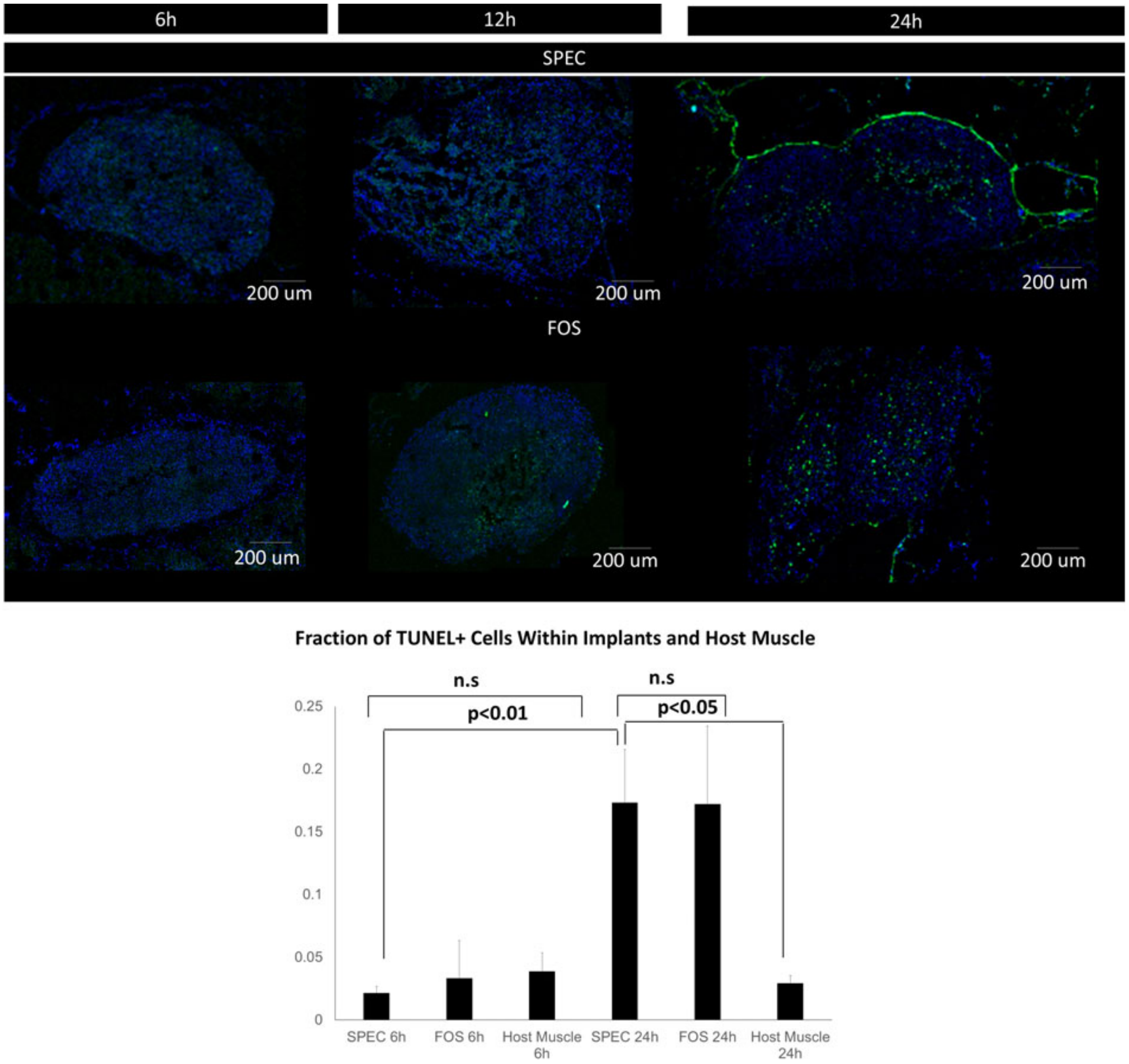

SUPPLEMENTARY FIG. S2. SPEC and FOS sections (10 μm) harvested at 6, 12, and 24 h were stained with Tdt dUTP Nick-End Labeling Assay (green) and counterstained with Hoechst nuclear stain (blue). A significant increase in TUNEL$^+$ cells was observed between 6 and 24 h for FOS and SPEC implants ($n = 4$, $p < 0.01$) compared to the surrounding rat host muscle. FOS, fibroblast-only spheroids; ns, no statistical significance.
