# Supplementary material for: Vascular Tissue Engineering Using Scaffold-Free Prevascular Endothelial–Fibroblast Constructs
Source: Biores Open Access. 2019 Jan 8;8(1):1–15. doi: 10.1089/biores.2018.0039 (PMC6327854; doi:10.1089/biores.2018.0039)
Supplement: Supplemental data [file Supp_Fig3.pdf]

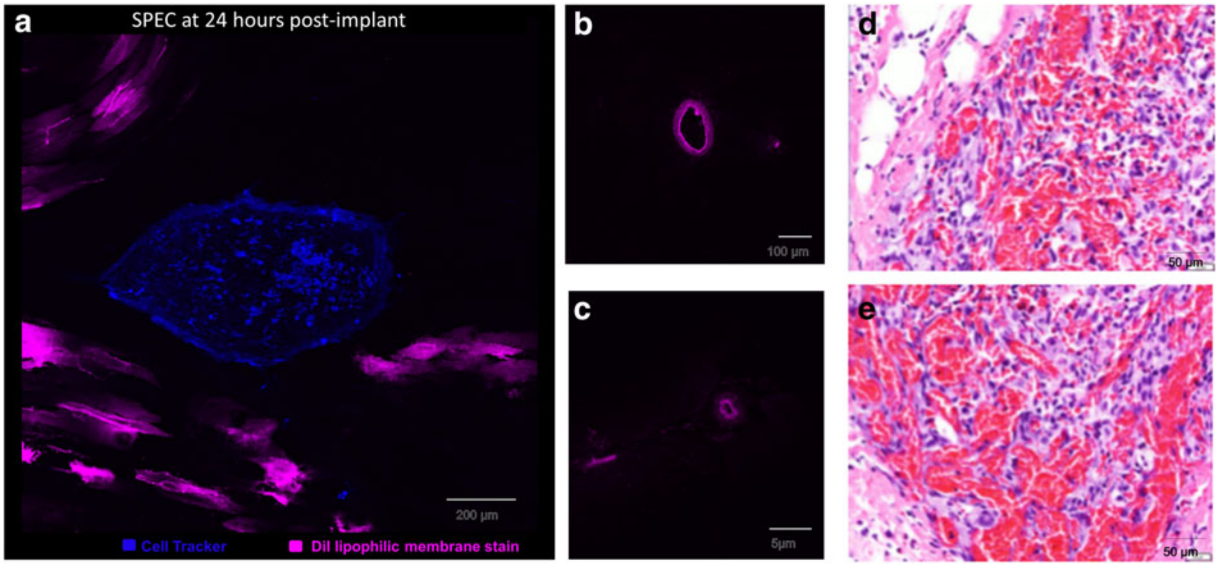

SUPPLEMENTARY FIG. S3. Rat hosts were prepared for perfusion 24 h postimplantation. Hosts were perfused with DiI lipophilic membrane stain (120 μg/mL in PBS with 5% glucose) following puncture of the left ventricle at a rate of 2 mL per min. DiI-perfused vessels were seen through confocal microscopy (purple). The implants were visualized by Deep Red Cell Tracker (blue). Vessels in the muscle tissue surrounding the implants **(a)** and distal to the implants on the opposite host hind limb **(b, c)** were clearly labeled with DiI. DiI successfully perfused and stained vessels of arteriole/venule (100–300 μm) diameters **(b)** or capillary (5–10 μm) diameters **(c)**. **(d, e)** Hematoxylin and eosin stains of implants harvested at 24 h, however, contain red blood cells and evidence of leaky appearing vasculature.
